# Organic contaminants in Ganga basin: from the Green Revolution to the emerging concerns of modern India

**DOI:** 10.1016/j.isci.2021.102122

**Published:** 2021-02-03

**Authors:** Aurora Ghirardelli, Paolo Tarolli, Mangalaa Kameswari Rajasekaran, Amogh Mudbhatkal, Mark G. Macklin, Roberta Masin

**Affiliations:** 1 Department of Agronomy, Food, Natural resources, Animals and Environment, University of Padova, Agripolis, Viale dell'Università 16, Legnaro, PD 35020, Italy; 2 Department of Land, Environment, Agriculture and Forestry, University of Padova, Agripolis, Viale dell'Università 16, Legnaro, PD 35020, Italy; 3 Ministry of Earth Sciences, Government of India, New Dehli 110003, India; 4 Lincoln Centre for Water and Planetary Health, School of Geography, College of Science, University of Lincoln, Brayford Pool, Lincoln, Lincolnshire LN6 7TS, UK; 5 Innovative River Solutions, School of Agriculture and Environment, Massey University, Palmerston North 4442, New Zealand; 6 Centre for the Study of the Inland, La Trobe University, Melbourne Campus, Bundoora VIC 3086, Australia

**Keywords:** Earth Sciences, Environmental Science, Environmental Monitoring, Pollution

## Abstract

The Ganga basin includes some of the most densely populated areas in the world, in a region characterized by extremely high demographic and economic growth rates. Although anthropogenic pressure in this area is increasing, the pollution status of the Ganga is still poorly studied and understood. In the light of this, we have carried out a systematic literature review of the sources, levels and spatiotemporal distribution of organic pollutants in surface water and sediment of the Ganga basin, including for the first time emerging contaminants (ECs). We have identified 61 publications over the past thirty years, with data on a total of 271 organic compounds, including pesticides, industrial chemicals, and by-products, artificial sweeteners, pharmaceuticals, and personal care products (PPCPs).

The most studied organic contaminants are pesticides, whereas knowledge of industrial compounds and PPCPs, among which some of the major ECs, is highly fragmentary. Most studies focus on the main channel of the Ganga, the Yamuna, the Gomti, and the deltaic region, while most of the Ganga's major tributaries, and the entire southern part of the catchment, have not been investigated. Hotspots of contamination coincide with major urban agglomerations, including Delhi, Kolkata, Kanpur, Varanasi, and Patna. Pesticides levels have decreased at most of the sites over recent decades, while potentially harmful concentrations of polychlorinated biphenyls (PCBs), organotin compounds (OTCs), and some PPCPs have been detected in the last ten years. Considering the limited geographical coverage of sampling and number of analyzed compounds, this review highlights the need for a more careful selection of locations, compounds and environmental matrices, prioritizing PPCPs and catchment-scale, source-to-sink studies.

## Graphical abstract

**Figure:**
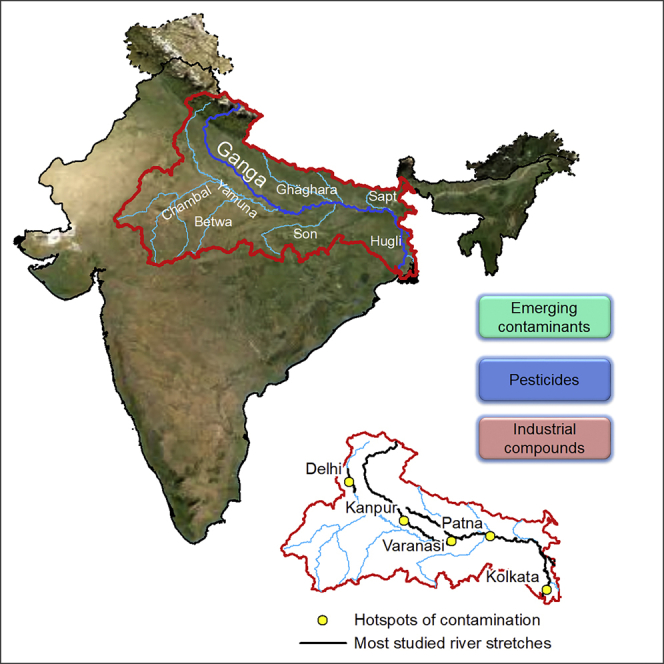

## Introduction

In recent decades, pollution of water bodies has become a matter of growing concern in the low- and middle-income countries. Rapid industrialization and population growth have increased the release of industrial and domestic effluents to surface water, jeopardizing aquatic ecosystems and compromising water quality (Paul, 2017).

The Ganga basin, one of the most densely populated areas in the world with exceptionally high population and economic growth rates (Census Data, 2011), is typical in this respect where the widespread contamination of water bodies has become a growing concern. Sediment and water carried by the Ganga and its tributaries represent a crucial resource for agriculture and many other economic activities, directly or indirectly supporting the livelihood of over 400 million people (Kumar, 2017).

Despite growing anthropogenic pressure in the catchment and severe water quality deterioration (Dwivedi et al., 2018), the pollution status of the Ganga is still poorly studied. Recent reviews have been either general summaries of pollution in the Ganga (Agarwal, 2015; Dwivedi et al., 2018), only reporting the main sources of contamination and not analyzing concentration trends, or broader studies about the Indian context that do not consider the river basin as an independent hydrological unit (Agarwal et al., 2015; Balakrishna et al., 2017; Chakraborty et al., 2014; Mathew and Kanmani, 2020; Mohapatra et al., 1995; Philip et al., 2018).

The main sources of contamination in the Ganga and its tributaries are sewage, industrial effluent, agricultural runoff, and religious activities (Dwivedi et al., 2018). Several researchers have reviewed the status of heavy metal residues in water and sediment (Paul, 2017; Singh Sankhla et al., 2018), while the total organic carbon and the presence of coliforms are regularly monitored by the Indian authorities for public health reasons (CPCB, 2013). However, less attention has been given to most classes of organic compounds, both synthesized intentionally and formed as by-products of human activities. Previous reviews generally focused on specific categories of contaminants (Goel et al., 2013; Sinha and Loganathan, 2015).

In the light of this, we review in this paper the environmental status of the Ganga and its tributaries in India, with particular reference to the spatiotemporal distribution of organic contaminants at a basin scale. In addition to pesticides and common industrial compounds, this study includes a specific focus on emerging contaminants (ECs) such as antibiotics, nonsteroidal anti-inflammatory drugs (NSAIDs) and artificial sweeteners (ASWs), which to our knowledge have never been systematically reviewed in the Ganga basin. We identify pollution hotspots as well as knowledge gaps, in order to guide future research campaigns and management policies that need to be implemented in the basin.

## Study area

The Ganga basin is the largest catchment within the Indian sub-continent (NMCG, 2012), covering an area of 1.086 million km^2^ (CPCB, 2013), 79 per cent of which is in located in India (Mirza, 2004). The Ganga originates from Gangotri glacier near Gomukh (Uttarakhand) where the Bhagirathi river begins at an elevation of about 7010 m above mean sea level. The combined flow formed at the confluence between the Bhagirathi and the Alaknanda, is known by the name Ganga (Sinha, 2004). After flowing for over 2525 km through the plains of Uttarakhand, Uttar Pradesh, Bihar, Jharkhand, and West Bengal, the Ganga discharges into the Bay of Bengal. The Indian section of the Ganga delta conventionally begins after the Farakka barrage, close to the border between India and Bangladesh. Downstream of the barrage, the final reach of the main channel is known as Hugli (Jain et al., 2007). Along its course, the Ganga is joined by many tributaries, the longest of which is the Yamuna, which crosses the National Capital Territory (NCT, Delhi).

Water flow in the river system is highly seasonal due to the Indian Summer Monsoon: about 84 per cent of the total rainfall occurs in the monsoon season, from June to September (CWC, 2014).

With its 450 million inhabitants, the Ganga basin is one of the most populous regions on Earth (Kumar, 2017). According to the *2011 Census Data*, the average population density in the Ganga basin is 520 persons per square kilometer, as compared to 312 for the rest of India. In the delta zone, the average population density rises to over 900 people per square kilometer. Since the mid-20^th^ century, the population of the eleven Indian states comprising the Ganga basin has grown considerably from 170 million people in 1951 to 611 million people in 2011 (Census Data, 2011). In the 21^st^ century, demographic growth has particularly affected urban areas, where population increased by 30 per cent between 2001 and 2011.

The Ganga basin is also the primary contributor to the agricultural economies of India, thanks to the availability of fertile soils across the region (NMCG, 2011). As a consequence, more than 65 per cent of the basin area is covered with agricultural land (CWC, 2014). Besides agriculture, hundreds of industrial plants are situated in the basin, comprising thermal power plants, electric industries, textiles, wood and jute mills, sugar mills, distilleries, pulp and paper factories, synthetic rubber industries, dairies, coal washeries, pesticide factories, and tanneries (Dwivedi et al., 2018). The major industrial centers of the basin, with around 1000 production units, are located in Uttar Pradesh. The biggest industrial cities are concentrated in the area from Kannauj to Varanasi: the leading economic activities in Kanpur, Allahabad, and Varanasi are focused on tannery, engineering, carpets, and locomotive sectors (Dwivedi et al., 2018).

Two of the world's largest industrial cities, Kolkata and Delhi, with 14.0 million and 16.35 million inhabitants, respectively, are located in the Ganga basin (Census Data, 2011).

### Organization of the database and selected bibliography

Only articles whose study area fell within the watershed of the Ganga (as defined by India Water Resources Information System (CWC, 2014), Figure 1) were considered in this review. Primary data related to river sediment and surface water were selected, the latter comprising river, pond, artificial canal and reservoir water bodies. A total of 61 papers provided primary data on the occurrence of organic contaminants in surface water and river sediment. Of these, 28 publications assessed surface water quality, 21 sediment and 12 analyzed both water and sediment. Besides the Ganga itself, most of the sampling areas are located along the Yamuna, the Gomti, and the delta (Hugli reach) (Figures 2A–2C), coinciding with big urban agglomerations such as Delhi, Kanpur, Allahabad, Varanasi, Patna, and Kolkata. The time period of this review covers the last 33 years, from 1986 (when the earliest analyzed paper was published) to 2019. Fifty of 61 articles were published after 2000, showing a growing interest in Indian environmental issues in the new millennium.

**Figure 1:**
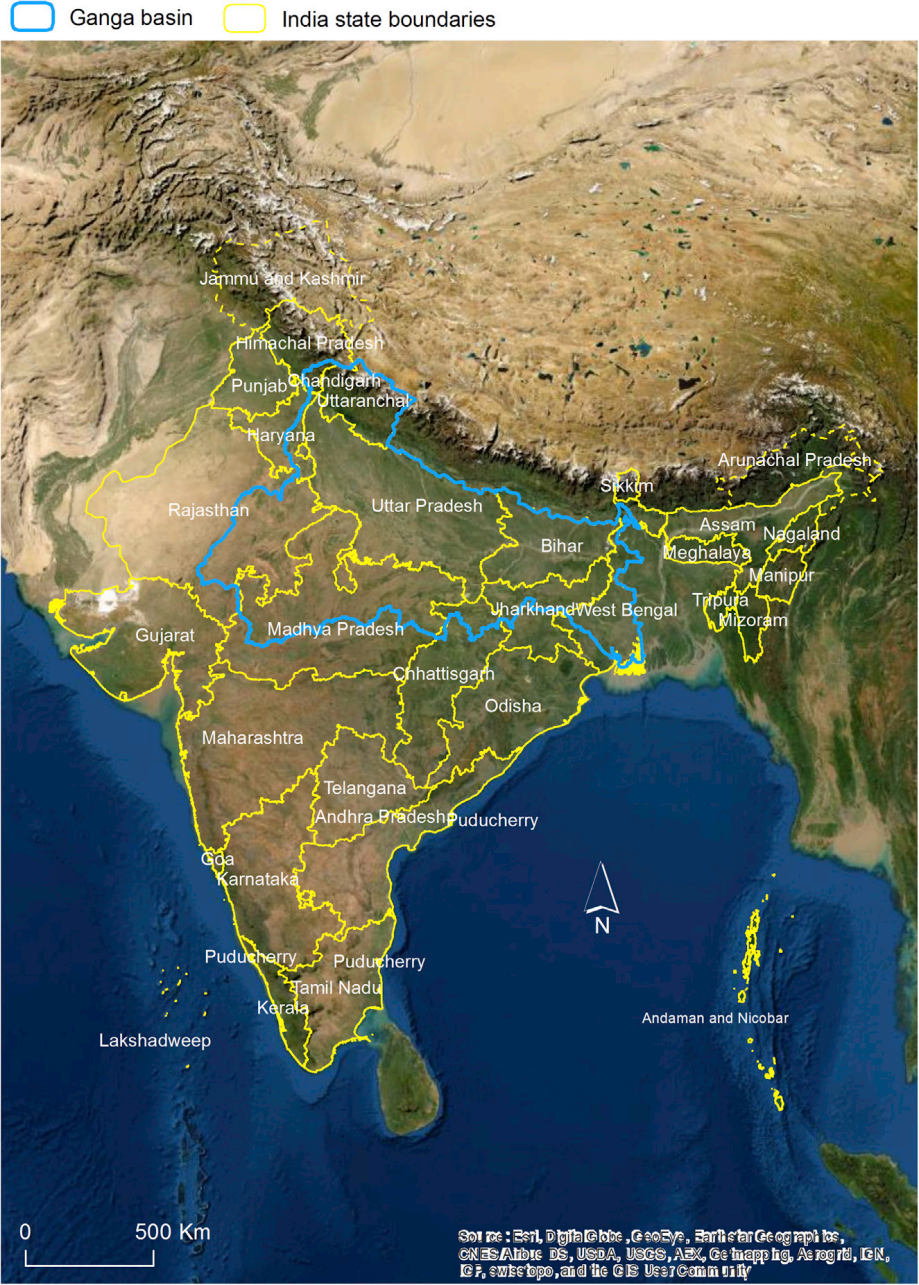
Study area and state boundaries within the Indian section of the Ganga basin

**Figure 2:**
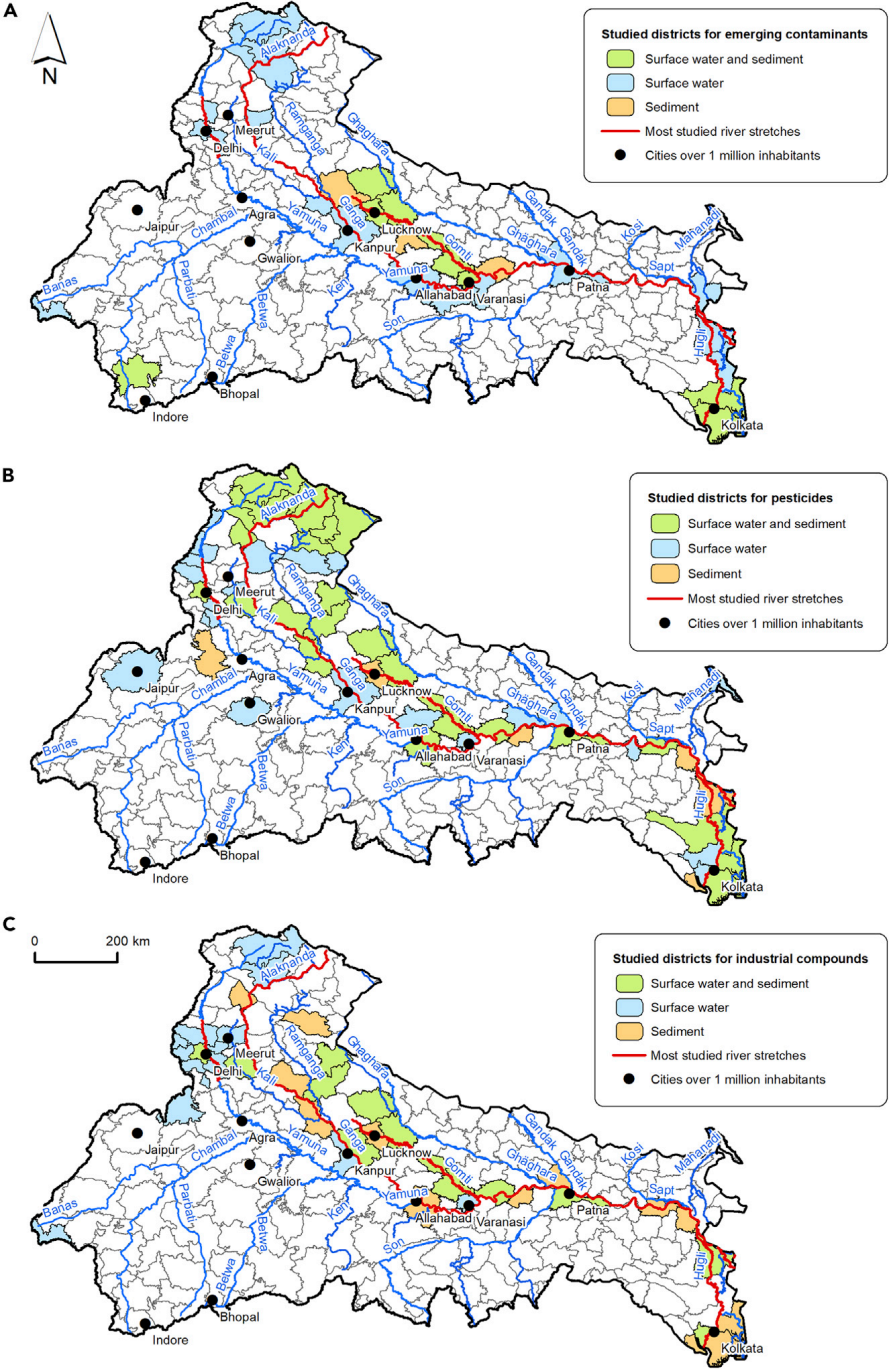
Spatial distribution of the studied districts within the Ganga basin (A) Emerging contaminants in surface water and sediment. (B) Pesticides in surface water and sediment. (C) Industrial compounds in surface water and sediment.

A total of 261 individual organic compounds and 10 groups of compounds (detected as cumulative concentrations) are reported, and these are classified into three broad categories: ECs (including pharmaceuticals, PCPs, caffeine, ASWs, parabens, phthalate plasticizers, benzotriazoles, bisphenol A, and PFAS), pesticides (including organochlorine pesticides (OCPs), organophosphates (OPhs), pyrethroids, herbicides, and fungicides) and industrial compounds (including polychlorinated biphenyls (PCBs), polybrominated diphenyl ethers (PBDEs), organotin compounds (OTCs), and polycyclic aromatic hydrocarbons (PAHs)).

### Emerging contaminants

According to the definition provided by the United States Geological Survey (USGS), ECs are “Any synthetic or naturally occurring chemical or any microorganism that is not commonly monitored in the environment but has the potential to enter the environment and cause known or suspected adverse ecological and/or human health effects” (Churchill et al., 2020; Philip et al., 2018). Many substances used in daily life, ranging from pharmaceuticals to detergents fall under this description (Philip et al., 2018; Sharma and Kapoor 2014; Stuart et al., 2012).

Within pharmaceuticals, antibiotics are receiving increasing attention because of their ability to induce the development of antibiotic resistance in pathogenic bacteria (Kümmerer, 2009). Besides antibiotics, NSAIDs (e.g. diclofenac and ibuprofen) and other drugs such as acetaminophen (paracetamol) and carbamazepine (an anti-epileptic compound), are emerging as possible threats to aquatic ecosystems. Their effects on biota range from physiological to behavioral alterations (Brodin et al., 2014; Klimaszyk and Rzymski, 2018). In addition, NSAIDs are known for their toxicity on avian species, first reported in scavenger birds of the Indian sub-continent (Cuthbert et al., 2007; Naidoo et al., 2010). Also PCPs, employed as active substances or preservatives in cosmetics, body care products, surfactants, detergents, insect repellents, and sunscreen agents have been widely studied in relation to their detrimental effects on aquatic biota (Champagne, 2009; Stuart et al., 2012) and antimicrobial resistance (Scientific Committee on Emerging and Newly Identified Health Risks, SCHENIR, 2009).

A major concern raised by the presence of pharmaceuticals and personal care products (PPCPs) in aquatic environments is their ability to interfere with the endocrine system, altering its normal functioning (Ebele et al., 2017). A primary example of such compounds, referred to as endocrine disruptors (World Health Organization and United Nations Environmental program, WHO and UNEP, 2013), are steroid hormones (Irwin et al., 2001; Jobling et al., 1998; Länge et al., 2001; Purdom et al., 1994; Tyler et al., 2005), whose presence in the aquatic environment can be related both to natural excretion and to synthetic estrogens and progestogens used in animal husbandry (Kuster et al., 2004) and for medical purposes (Monteiro and Boxall, 2010).

Besides PPCPs, other compounds have been widely reported to exhibit endocrine-disrupting properties, such as bisphenol A (Eladak et al., 2015; Rezg et al., 2014; Rochester, 2013; Vandenberg et al., 2012), an essential component of epoxy resins (Eladak et al., 2015), and phthalates, mainly employed as plasticizers (Petrović et al., 2001).

ASWs are one of the most recently recognized classes of high-priority ECs among non-PPCPs, as they are frequently detected in different environmental matrices (Luo et al., 2019). Saccharine, cyclamate, acesulfame K, and sucralose are the most studied compounds. Although their ecotoxicity is still poorly understood (Luo et al., 2019), they are viewed as ideal indicators of domestic wastewater contamination in surface and groundwater (Tran et al., 2014).

In this study, compounds have been included in the class of ECs based on literature definitions, but also on the basis that they are not yet included in routine monitoring campaigns in India, and that first recordings of these chemicals in the Ganga basin are very recent in comparison with pesticides and industrial compounds (ICs, Table 1, Table 2, Table 3, Table 4, Table 5, Table 6 and S4–S9).

**Table 1:** Summary table of compounds, study areas and maximum concentrations of emerging contaminants in Ganga basin surface water. Abbreviations are listed in Table S10.See also Table S4.

Compounds	Study area	Maximum concentration	ng/L	References
PFAS (20 compounds)	Ganga, Hugli	PFHxA	2.29	(Yeung et al., 2009)
Anionic surfactants	Hugli and small tributaries (Kolkata)	Total anionic surfactants	425,000	(Ghose et al., 2009)
NSAIDs, other pharmaceuticals	Yamuna (Delhi area)	–	–	(Mutiyar and Mittal, 2012)
Other compounds (caffeine)	Yamuna (Delhi area)	Caffeine	808	Mutiyar and Mittal (2012)
Antibiotics	Yamuna (Delhi area)	Ampicillin	27,100	(Mutiyar and Mittal, 2014a)
PFAS (21 compounds)	Bhagirathi, Alaknanda and Ganga	PFBS	10.2	(Sharma et al., 2016)
NSAIDs, other pharmaceuticals	Yamuna (Delhi area)	Ibuprofen	2302	(Mutiyar et al., 2018)
Other compounds (caffeine)	Yamuna (Delhi area)	Caffeine	2640	(Mutiyar et al., 2018)
Antibiotics	Kshipra (Ujjain)	Sulfamethoxazole	4660	(Diwan et al., 2018)
Biocides (triclosan)	Gomti	Triclosan	9650	(Nag et al., 2018)
Antibiotics, NSAIDs, other pharmaceuticals	Bhagirathi, Alaknanda and Ganga	Ketoprofen	107	(Sharma et al., 2019)
Insect repellent products, biocides (DEET, triclocarban, triclosan)	Bhagirathi, Alaknanda and Ganga	DEET	22.3	(Sharma et al., 2019)
Artificial sweeteners	Bhagirathi, Alaknanda and Ganga	Saccharine	85.43	(Sharma et al., 2019)
Other compounds (caffeine)	Bhagirathi, Alaknanda and Ganga	Caffeine	743	(Sharma et al., 2019)
Antibiotics, NSAIDs, other pharmaceuticals	Ahar, Pichola Lake and Fateh Sagar Lake (Udaipur)	Caffeine	37,476	(Williams et al., 2019)
Hormones	Ahar, Pichola Lake and Fateh Sagar Lake (Udaipur)	Androsterone	1557	(Williams et al., 2019)
Insect repellent products, biocides (DEET, triclocarban, triclosan)	Ahar, Pichola Lake and Fateh Sagar Lake (Udaipur)	DEET	388	(Williams et al., 2019)
Other compounds (bisphenol A, benzotriazole, methylbenzotriazole, caffeine)	Ahar, Pichola Lake and Fateh Sagar Lake (Udaipur)	Caffeine	37,476	(Williams et al., 2019)

**Table 2:** Summary table of compounds, study areas, and maximum concentrations of emerging contaminants in Ganga basin river sediment. Abbreviations are listed in Table S10. See also Table S5.

Compound	Study area	Maximum concentration	μg/kg d.w.	References
Phtalates	Gomti	DEHP	324.72	(Srivastava et al., 2010)
PFAS (PFOA, PFOS)	Hugli, Sundarban wetland	PFOA	14.09	(Corsolini et al., 2012)
Antibiotics	Kshipra (Ujjain)	Ofloxacin	9.74	(Diwan et al., 2018)
Biocides (triclosan)	Gomti	Triclosan	50.35	(Nag et al., 2018)
NSAIDs, other pharmaceuticals	Hugli	Carbamazepine	519	(Chakraborty et al., 2019)
Biocides (triclosan), musk fragrances, Preservatives (parabens)	Hugli	Methyl paraben	423	(Chakraborty et al., 2019)
Other compounds (bisphenol A, phtalates, DEHA)	Hugli	DEHP	300	(Chakraborty et al., 2019)

**Table 3:** Summary table of compounds, study areas and maximum concentrations of pesticides in Ganga basin surface water. Abbreviations are listed in Table S10. See also Table S6.

Compound classes	Study area	Maximum concentration	ng/L	References
OCPs	Yamuna (Delhi area)	p,p'-DDT	1610	(Agarwal et al., 1986)
OCPs	Mahala water reservoir (Jaipur)	γ-HCH	26,360	(Bakre et al., 1990)
OCPs	Yamuna (Delhi area)	Dieldrin	100,000	(Nair et al., 1991)
OCPs	Ganga (Varanasi)	p'-DDT	79,818	(Nayak et al., 1995)
OCPs; herbicides; OPhs	Ganga (Kachla to Kannauj)	p,p'-DDT	5330	(Rehana et al., 1995)
OCPs; herbicides; OPhs	Ganga (Narora)	α-HCH	1380	(Rehana et al., 1996)
OCPs	22 ponds (Shahjahanpur)	β-HCH	10,110	(Dua et al., 1996)
OCPs	7 Himalayan lakes (Nainital region)	p,p'-DDT	22,222	(Dua et al., 1998)
OCPs	Rivers and streams of the Kumaun Himalayan region	Total DDT	9072	(Sarkar et al., 2003)
OCPs; OPhs	Ganga (Kanpur)	Malathion	2610	(Sankararamakrishnan et al., 2005)
OCPs; herbicides; OPhs	Yamuna (Delhi area)	Total endosulfan	114	(Aleem and Malik, 2005)
OCPs	Bhagirathi, Alaknanda, Ganga and minor rivers of Uttarakhand	Total DDT	364.81	(Semwal and Akolkar, 2006)
OCPs	Streams, ponds and canals between Kanpur and Lucknow	β-HCH	1320	(Singh et al., 2007)
OCPs	Yamuna and canals (Delhi and Haryana)	p,p'-DDT	1423.44	(Kaushik et al., 2008)
OCPs	Gomti	β-HCH	301.44	(Malik et al., 2009)
OCPs	Hugli and small tributaries (Kolkata)	Other HCH isomers	7820	(Ghose et al., 2009)
OCPs; OPhs; pyrethroids	flowing water bodies adjacent to the tea gardens of Dooars and Hill regions	Heptachlor	7.6	(Bishnu et al., 2009)
OCPs	Sharda river, Reetha river, drains surrounding lindane factory (Lucknow)	α-HCH	290,000	(Jit et al., 2011)
OCPs; OPhs	Ganga and Jamania river (Bhagalpur)	α-endosulfan	739	(Singh et al., 2012)
OCPs	Yamuna (Delhi area)	p,p'-DDT	239	(B. Kumar et al., 2012b)
OCPs	Ganga and tributaries in upper, middle and lower reach	Total endosulfsn	17.9	(Mutiyar and Mittal, 2013)
OCPs	Ganga and Yamuna (Allahabad)	γ-HCH	24,500	(Raghuvanshi et al., 2014)
OCPs; OPhs	Tighra reservoir (Gwalior)	Dichlorvos	22.3	(Rao and Wani, 2015)
OCPs; herbicides	Gomti	Buthachlor	135,000	(Trivedi et al., 2016)
OCPs; OPhs; herbicides; fungicides	Hugli	δ-HCH	2940	(Mondal et al., 2018)

**Table 4:** Summary table of compounds, study areas and maximum concentrations of pesticides in Ganga basin river sediment. Abbreviations are listed in Table S10. See also Table S7.

Compound classes	Study area	Maximum concentration	μg/kg d.w.	References
OCPs	Yamuna (Delhi area)	p,p'-DDT	3060	(Agarwal et al., 1986)
OCPs	22 ponds (Shahjahanpur)	o,p'-DDT	908.25	(Dua et al., 1996)
OCPs	Ganga (Narora to Kannauj)	Heptachlor epoxide	18	(Ahmad et al., 1996)
OCPs	Ganga and tributaries in upper, middle and lower reach	Chlordane + metabolites	49	(Senthilkumar et al., 1999)
OCPs	Hugli	Endosulfan sulfate	400	(Bhattacharya et al., 2003)
OCPs	Hugli, Sundarban wetland	p,p'-DDT	1.29	(Guzzella et al., 2005)
OCPs	Bhagirathi, Alaknanda, Ganga and minor rivers of Uttarakhand	Not detected	–	(Semwal and Akolkar, 2006)
OCPs	Hugli, Sundarban wetland	p,p'-DDT	8.48	(Sarkar et al., 2008)
OCPs	Gomti	o,p'-DDT	345.66	(Malik et al., 2009)
OCPs	Yamuna (Delhi area)	Endrin aldehyde	90.87	(Pandey et al., 2011)
OCPs	Drains discharging into Yamuna (Delhi area)	Chlorpyriphos	286.56	(Kumar et al., 2011)
OCPs; OPhs	Ganga and Jamania River (Bhagalpur)	p,p'-DDT	3329.3	(Singh et al., 2012)
OCPs	Wetlands in Keoladeo National Park	γ-HCH	7540	(Singh et al., 2012)
OCPs	Ganga and Yamuna (Allahabad)	γ-HCH	19.8	(Raghuvanshi et al., 2014)
OCPs; OPhs; herbicides; fungicides	Hugli	δ-HCH	0.987	(Mondal et al., 2018)

**Table 5:** Summary table of compounds, study areas and maximum concentrations of industrial compounds in Ganga basin surface water. Abbreviations are listed in Table S10. See also Table S8.

Compound classes	Study area	Maximum concentration	ng/L	References
OTCs (dimethyltin, monobutyltin, dibutyltin, tributyltin)	Ganga, Pandu, Loni and Ganda Nala rivers (Kanpur-Unnao)	MBT	70.1 (ng Sn/L)	(Ansari et al., 1998)
PAHs (16 compounds)	Gomti	Acenaphthylene	65,850	(Malik et al., 2004)
PAHs (16 compounds)	Gomti	Acenaphthylene	82,670	(Malik et al., 2011)
OTCs (monobutyltin, dibutyltin, tributyltin)	Kolkata harbor	DBT	104 (ng Sn/L)	(Garg et al., 2011)
PCBs (28 congeners)	Yamuna and canals, lakes, ponds and drains (Delhi area)	PCB-44	594	(S. Kumar et al., 2012)
PCBs (27 congeners)	Yamuna (Delhi area)	PCB-18	280	(B. Kumar et al., 2012b)
PAHs (16 compounds)	Bhagirathi, Alaknanda and Ganga	Pyrene	21.21	(Sharma et al., 2018)

**Table 6:** Summary table of compounds, study areas, and maximum concentrations of industrial compounds in Ganga basin river sediment. Abbreviations are listed in Table S10. See also Table S9.

Compound classes	Study area	Maximum concentration	μg/kg d.w.	References
PAHs (benzo[a]pyrene, phenantrene)	Ganga (Narora to Kannauj)	Phenantrene	18	(Ahmad et al., 1996)
Total PCBs	Ganga and tributaries in upper, middle and lower reach	Total PCBs	15	(Senthilkumar et al., 1999)
PAHs (16 compounds)	Gomti	Benzo[a]anthracene + chrysene	1569.94	(Malik et al., 2004)
PAHs (19 compounds)	Hugli, Sundarban wetland	Fluoranthene	214	(Guzzella et al., 2005)
PCBs (13 congeners); PAHs (19 compounds)	Hugli, Sundarban wetland	PCB-153	0.54	(Guzzella et al., 2005)
PAHs (16 compounds)	Yamuna (Delhi area)	Naphtalene	4610	(Agarwal et al., 2006)
PBDEs (12 congeners)	Hugli, Sundarban wetland	PBDE-47	8.832	(Binelli et al., 2007)
Total PAHs (19 compounds)	Hugli, Sundarban wetland	Total PAHs	4249.71	(Binelli et al., 2008)
PCBs (23 congeners)	Hugli, Sundarban wetland	PCB-138	6.08	(Binelli et al., 2009)
PAHs (16 compounds)	Hugli, Sundarban wetland	Acenaphthylene	1521	(Tripathi et al., 2009)
Total PAHs (10 compounds)	Nainital and Bhimtal Lakes	Total PAHs	217,000	(Choudhary and Routh, 2010)
PAHs (16 compounds)	Gomti	Acenaphthylene	2726.4	(Malik et al., 2011)
OTCs	Hugli, Sundarban wetland	TBT	84.2	(Antizar-Ladislao et al., 2011)
OTCs	Kolkata harbor	TBT	442 (ng Sn/g)	(Garg et al., 2011)
PCBs (28 congeners)	Yamuna (Delhi area)	PCB-44	14.17	(B. Kumar et al., 2012b)
PBDEs (22 congeners)	Canals in Kolkata	PBDE-47	0.615	(Kwan et al., 2013)
PAHs (16 compounds)	Hugli, Sundarban wetland	Fluoranthene	1839.5	(Zuloaga et al., 2013)

### Location of sample points

Only 13 papers evaluate ECs, but the majority of them analyzed simultaneously different sub-categories, including PPCPs, with the prevalence of biocides, antibiotics, and NSAIDs.

The sample points for PPCPs are all concentrated in the main channel of the river system (i.e., especially around the cities of Kanpur, Allahabad, Varanasi, and Patna, located in the middle reach of the Ganga basin, and in the Hugli and deltaic region (Figure 2A and Tables 1 and 2). Besides the main channel, papers mainly focused on the NCT in the Yamuna sub-catchment. In addition, Sharma et al. (2019) also monitored the rivers Alaknanda and Bhagirathi in the Himalayan reach. The remaining publications focused on the cities along the Gomti river (Nag et al., 2018), in Ujjain (Madhya Pradesh) (Diwan et al., 2018) and Udaipur (Rajasthan) (Williams et al., 2019).

However, the distribution pattern for surface water sampling differs considerably from sediment sampling areas: papers reporting on water pollution were focused on big urban agglomerates such as Delhi (Mutiyar et al., 2018; Mutiyar and Mittal, 2012, 2014a), Kanpur, Allahabad, Varanasi, Patna (Sharma et al., 2019), and Lucknow (Nag et al., 2018). In the case of sediment, addressed only by Nag et al. (2018), Diwan et al. (2018), and Chakraborty et al. (2019), sample points were located along the Gomti river, in the city of Ujjain, and the Hugli area.

Some of the PCPs have been evaluated by only one paper, and in restricted reaches of the basin: synthetic detergents (anionic surfactants) in sediment samples collected in Kolkata district (Ghose et al., 2009), musk fragrances and parabens in sediment along the Hugli (Chakraborty et al., 2019).

With regard to non-PPCP compounds, the presence of ASWs has been reported only by Sharma et al. (2019) in the main channel and the Himalayan rivers, whereas benzotriazole and methylbenzotriazole have been reported by Williams et al. (2019) near Udaipur.

The distribution of bis (2- ethylhexyl) adipate plasticizers has been studied by Chakraborty et al. (2019) along the Hugli, while phthalates have been assessed in sediment both in the Gomti (Srivastava et al., 2010) and the Hugli (Chakraborty et al., 2019). Bisphenol A has been studied both by Chakraborty et al. (2019) in the Hugli and by Williams et al. (2019) in surface water near Udaipur. Levels of PFAS has been evaluated by three papers: Yeung et al. (2009) and Sharma et al. (2016) focused on water samples from cities and towns located along the main channel, the Alaknanda, the Bhagirathi and the confluence between Ganga and Yamuna; Corsolini et al. (2012) studied sediment contamination in the Hugli river and adjacent Sundarban wetlands.

### Occurrence of ECs

The maximum concentrations of PPCPs in water exhibited a wide range from less than one to thousands of ng/L, while sediment concentrations varied between less than one and hundreds of μg/kg.

With regard to pharmaceuticals, the compound with the highest water concentration was the antibiotic ampicillin (maximum recorded value, MRV: 27,100 ng/L, Delhi (Mutiyar and Mittal, 2014b)). For the NSAIDs ibuprofen had the highest values (MRV: 2302 ng/L, Delhi (Mutiyar et al., 2018)), and in the hormone group, the highest concentration reported was for androsterone (MRV: 1557 ng/L, Udaipur (Williams et al., 2019)). The PCP with the highest concentration in water was triclosan (MRV: 9650 ng/L, Gomti river (Nag et al., 2018)). The only reported value for surfactants was a cumulative concentration comprising all the methylene-blue-active substances: not surprisingly, it was higher than any single compound among PPCPs (MRV: 0.425 mg/L (425,000 ng/L), Kolkata (Ghose et al., 2009)).

Only six antibiotics, three NSAIDs, carbamazepine, musk ketone, four parabens, and triclosan have been assessed in river sediment (Tables 2 and S5). The highest recorded concentration for pharmaceuticals was 519 μg/kg dry weight (d.w.) (carbamazepine), followed by the NSAID ibuprofen (MRV: 340 μg/kg d.w., Hugli river (Chakraborty et al., 2019)); the MRV for antibiotics was 9.74 μg/kg d.w. (Ujjain (Diwan et al., 2018)). The highest PCP value was recorded for methyl paraben (MRV: 423 μg/kg d.w (Chakraborty et al., 2019)), whereas triclosan and musk ketone showed much lower concentrations (MRVs: 84 and 26 μg/kg d.w. respectively (Chakraborty et al., 2019)).

The highest concentration of non-PPCPs in water was found for caffeine (maximum recorded value, MRV: 37,476 ng/L, Udaipur (Williams et al., 2019)), and the highest sediment concentration was detected for di-(2-ethylhexyl) phthalate (MRV: 400 μg/kg d.w.), in the Hugli river (Chakraborty et al., 2019)). ASW maximum water concentrations were extremely low compared to other PPCPs sub-categories: the highest recorded value was 85.43 ng/L, found in Patna for saccharine (Sharma et al., 2019). In the case of PFAS, the highest water concentration was found for perfluorobutane sulfonate (PFBS) (MRV: 10.19 ng/L, Gangasagar (Sharma et al., 2016)), whereas the MRV for sediment was 14.09 μg/kg d.w. (PFOA, Sundarban wetland (Corsolini et al., 2012)).

**Figure 3:**
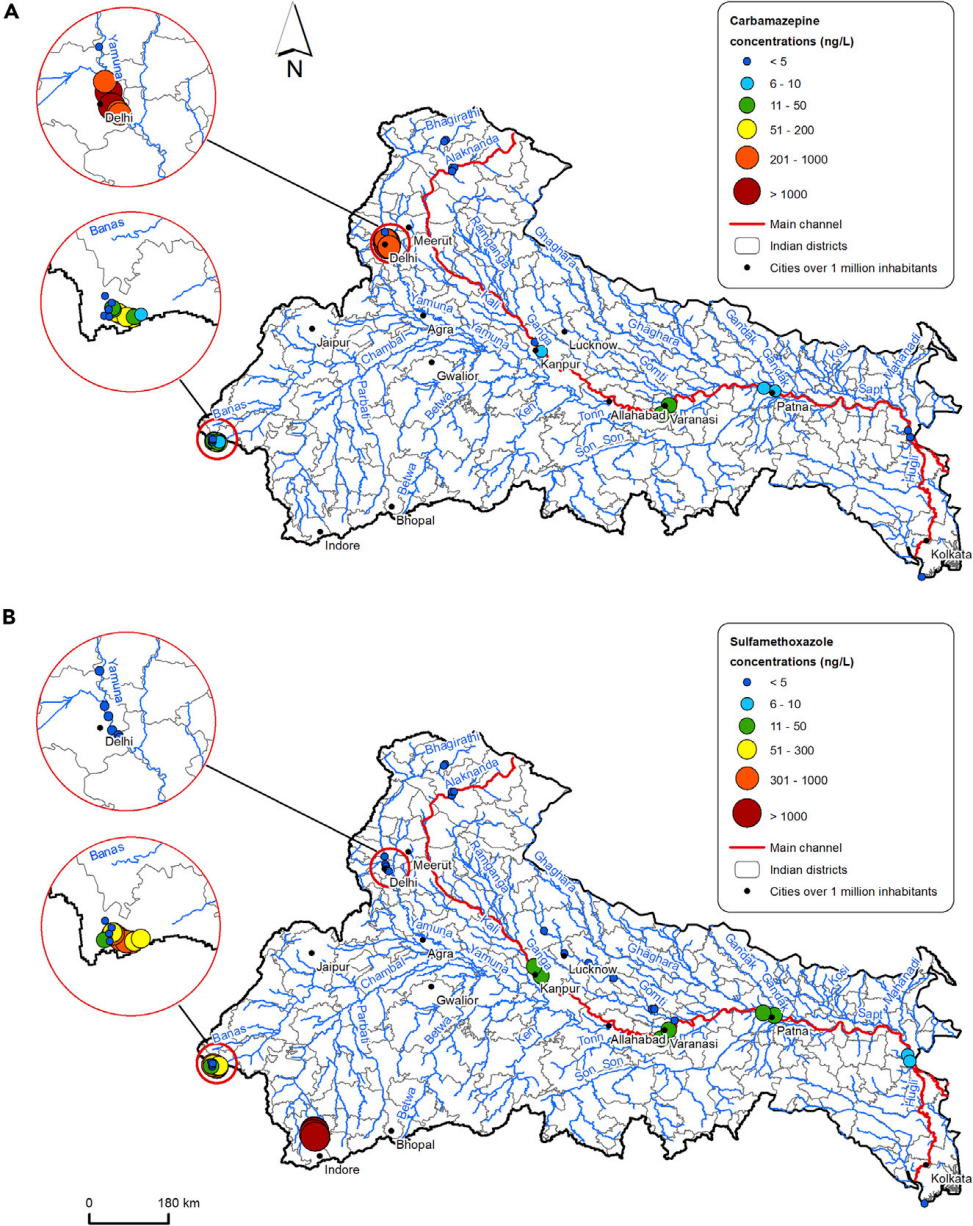
Maximum water concentrations of two selected ECs in the Ganga basin (A) Carbamazepine. (B) Sulfamethoxazole.

With regard to the spatial distribution, PPCPs analyzed by more than one paper, in different areas of the basin, are characterized by a wide range of variability in river water. Concentrations in the main channel and in headwater rivers are generally below 10 ng/L and often close to their limit of detection (usually 0.1–5 ng/L). Water concentrations tend to be considerably higher downstream of Delhi and in Udaipur: this pattern is evident for compounds such as acetaminophen, carbamazepine, ciprofloxacin, DEET, diclofenac (not detected in Delhi, but very high in Udaipur), hydrochlorothiazide, ibuprofen, naproxen, and sulfamethoxazole. The latter exhibited higher water concentrations also in Ujjain, and triclosan along the Gomti. Figure 3 shows the variations in levels of carbamazepine and sulfamethoxazole in the Ganga, selected to be representative of ECs.

Although the main Ganga channel was characterized by low concentrations (often below 10 ng/L), Sharma et al. (2019) recorded that the analyzed PPCPs were generally higher in middle and lower reaches compared to the Himalayan reach, most notably downstream of major cities such as Kanpur, Varanasi, and Patna. This pattern, also evident in compounds such as carbamazepine, hydrochlorothiazide, sulfamethoxazole, and diethyltoluamide (DEET, Figure S1), is likely to result from local releases of sewage and industrial wastewater, which are the main sources of ECs in water bodies. Pollution loads do not increase along the main channel (lower concentrations are recorded between Farakka and Gangasagar), and this behavior is likely to arise from natural attenuation processes (Narain, 2014). This has been observed on a smaller scale both in the Gomti (Nag et al., 2018) and the Kshipra (Diwan et al., 2018), where point sources predominate.

Similarly to what recorded by Sharma et al. (2019) for PPCPs, also Yeung et al. (2009) and Sharma et al. (2016) reported an increase in water concentration of PFAS up to the middle reach. The exception to this pattern is shown by caffeine, whose concentrations in the Himalayan reach of the Ganga were comparable to those detected in the middle and lower reaches of the basin (hundreds of ng/L). As a whole, its concentrations are generally higher than other ECs, due to the very large-scale consumption of this compound, which is common in food and beverages (Mutiyar et al., 2018).

As far as sediment concentrations are concerned, the few ECs for which more than one article was found showed comparable concentrations both along the Gomti (Nag et al., 2018; Srivastava et al., 2010) and the Hugli (Chakraborty et al., 2019): phthalates maximum concentrations were in the order of 300 ng/L, while triclosan maximum values ranged between 50 and 80 ng/L.

### Pesticides

Pesticides represent the most studied class of organic contaminants in the Ganga basin. They are a direct consequence of so called “Green Revolution”, which resulted the widespread adoption of new technologies and pesticides in agriculture in the 1970s.

The use of pesticides in modern agriculture has led to worldwide nonpoint pollution in aqueous environments, affecting water bodies used for drinking water (Schulz, 2004; Zhang and Zhang, 2011) and nontarget organisms (Barranger et al., 2014; Ma et al., 2006; Ogbeide et al., 2015; Stachowski-Haberkorn et al., 2013). However, pesticides can also originate from urban environments: in particular, household agents used for control of vector-borne diseases such as malaria or Leishmaniasis (Sinha and Loganathan, 2015).

In India, whose pesticide consumption accounts for just 3.75 per cent of worldwide use, 80 per cent is represented by insecticides, 15 per cent by herbicides, and 2 per cent by fungicides (Agarwal et al., 2015).

Although growing environmental and human health risks have led to worldwide bans of numerous pesticides (UNEP, 2018), they remain a matter of concern due to their high persistence and ubiquitous presence in ecosystems and the environment.

A prominent example of this is represented by OCPs, a class of insecticides and acaricides that include 9 of the first 12 contaminants listed in the Stockholm Convention on Persistent Organic Pollutants (UNEP, 2018). Like all Persistent Organic Pollutants (POPs), OCPs such as DDT and lindane are characterized by high hydrophobicity, lipophilicity, and persistence in the environment (Zitko, 2003), and tend to bioaccumulate in the fatty tissues of biota, especially at high trophic levels (Ntow et al., 2008). However, the use of DDT is still permitted in some regions of the world, including India, for applications against mosquitoes to control malaria. Similarly, lindane (γ-hexachlorocyclohexane, HCH isomer) can be employed for the control of body parasites (head lice and scabies) (UNEP, 2018).

OPhs are another class of insecticides and acaricides potentially harmful for a wide variety of non-target species (Goel et al., 2013) and responsible for frequent cases of human poisoning in India (Jokanović, 2018).

### Location of sample points

As a result of their wide use in the Ganga basin since the Green Revolution, pesticides are the most studied class of organic contaminants, with 33 papers reviewed in this study (Tables 3, 4, S6 and S7). The most frequently reported pesticides were OCPs, which have been documented along the entire course of the main channel and the upper reaches since the 1970s (Bakre et al., 1990). Study areas for pesticides are not homogeneously distributed in the Ganga basin; the majority are focused on the northern-central section of the basin, along the main channel, around cities such as Kanpur, Unnao, Allahabad, Varanasi, and Patna, and in the Himalayan reach of the Ganga (Alaknanda, Bhagirathi, and different streams). The Gomti sub-catchment, Delhi and the surrounding districts of Uttar Pradesh along the Yamuna, and the Hugli and Sundarban wetlands have also been investigated. To the best of our knowledge, south-bank rivers other than the Yamuna have not been assessed for pesticides (Figure 2B).

OCPs belonging to the DDT, endosulfan and HCH group (main isomers and related metabolites) and cyclodiene insecticides (aldrin, dieldrin, endrin) have been extensively analyzed in water and sediment along the entire course of the main channel and the rivers of the Himalayan region (Ahmad et al., 1996; Mutiyar and Mittal, 2013; Nayak et al., 1995; Raghuvanshi et al., 2014; Rehana et al, 1995, 1996; Sankararamakrishnan et al., 2005; Sarkar et al., 2003; Semwal and Akolkar, 2006; Senthilkumar et al., 1999; Singh et al., 2012). Studies on the Yamuna and its canals, however, were focused on the area around Delhi (Agarwal et al., 1986; Aleem and Malik, 2005; Kumar et al., 2011; B. Kumar et al., 2012b; Nair et al., 1991; Pandey et al., 2011) and the surrounding agricultural regions of Haryana, including Western Yamuna, Agra and Gurgaon canals (Kaushik et al., 2008). Only one paper has investigated the presence of the OCPs in the upper reach of Yamuna (Semwal and Akolkar, 2006). Water and sediment of the Hugli and Sundarban wetlands have been addressed by five papers ((Bhattacharya et al., 2003; Ghose et al., 2009; Guzzella et al., 2005; Mondal et al., 2018; Sarkar et al., 2008), the latter also sampling pond water). In addition, two studies assessed the presence of OCPs in water and sediment along the Gomti (Malik et al., 2009; Trivedi et al., 2016).

Bakre et al. (1990) investigated the presence of HCH, Heptachlor and Aldrin in the waters of Mahala reservoir, near Jaipur; Dua et al. (1996) focused on the distribution of DDT and HCH in 22 ponds in the district of Shahjahanpur, Uttar Pradesh; Dua et al. (1998) detected DDT and HCH compounds in Bhimtal, Sattal, Khurpatal, Naukuchiatal, and Nainital lakes (Nainital Himalayan region); Singh et al. (2007) addressed HCB and several compounds belonging to DDT, HCH, endosulfan, heptachlor, chlordane, and cyclodiene groups in streams, ponds, and canals between Kanpur and Lucknow; Bishnu et al. (2009) studied the presence of heptachlor, dicofol, and endosulfan in waterbodies adjacent to the tea gardens of Dooars and Hill regions, West Bengal; Singh Bhadouria et al. (2012) focused on compounds belonging to DDT, HCH, endosulfan, heptachlor, chlordane, and cyclodiene group, in the wetlands outside and inside Keoladeo National Park, Rajasthan; Rao and Wani (2015) investigated the presence of DDT, HCH, HCB, endosulfan, heptachlor, and cyclodiene pesticides in Tighra reservoir, near Gwalior (Madhya Pradesh). In addition, Jit et al. (2011), assessed the presence of HCH isomers in the Sharda and Reetha rivers, and in drains surrounding a lindane factory located in Lucknow district. The major contaminants in terms of records and spatial abundance of sample points were aldrin among the cyclodiene group, p,p’-DDT among the DDT group, γ-HCH among the HCH group, and both α- and β-endosulfan among the endosulfan group.

OPhs have only been detected around villages and cities located along the main channel, in the upper-middle reach, namely Kachla, Narora, Fatehgarh, Kannauj, and Kanpur (Rehana et al, 1995, 1996; Sankararamakrishnan et al., 2005). In addition, the presence of OPhs has been investigated in the Hugli river and the surrounding ponds by Mondal et al. (2018), and in the Yamuna by Aleem and Malik (2005) and Kumar et al. (2011), who exclusively focused on NCT.

Herbicides, such as alachlor, atrazine, and butachlor, have been studied in sediment, river, and pond water from four sites along the Hugli (Mondal et al., 2018), in drains discharging into the Yamuna in Delhi (Kumar et al., 2011), and along the Gomti, in the area of Lucknow (Trivedi et al., 2016). 2,4-dichlorophenoxyacetic acid (2,4-D) has been detected along the main channel in the upper-middle reach (Rehana et al, 1995, 1996) and NCT along the Yamuna (Aleem and Malik, 2005).

Both fungicides and pyrethroids have been detected only in the Hugli and in ponds of the deltaic region (Mondal et al., 2018), as well as in streams, ponds and canals between Kanpur and Lucknow (Bishnu et al., 2009).

### Occurrence of pesticides

Pesticides showed the greatest variability both in water and sediment, with values ranging from less than one ng/L to mg/L and from less than one to thousands of μg/kg d.w., respectively.

For OCPs, the compounds with the highest concentration were α-HCH for water (MRV: 0.29 mg/L (290000 ng/L) at Lucknow (Jit et al., 2011)) and γ-HCH for sediment (MRV: 7540 μg/kg d.w. at Bharatpur (Singh et al., 2012)).

Among OPhs, the highest water concentration was found for malathion (MRV: 2610 ng/L, Kanpur (Sankararamakrishnan et al., 2005)), whereas the MRV for sediment was 458.02 μg/kg d.w. (methyl parathion, Bhagalpur (Singh et al., 2012)).

The only available paper on pyrethroids (Bishnu et al., 2009) found concentrations below the detection limit. Similarly, the only analyzed fungicide (metalaxyl), was below the detection limit in sediment and 83 ng/L in water (final reach of the Hugli (Mondal et al., 2018)).

Herbicides with the highest concentration were butachlor in water (MRV: 0.135 mg/L (135000 ng/L), Lucknow (Trivedi et al., 2016)) and pendimethalin in sediment (MRV: 53.19 μg/kg d.w., Delhi (Kumar et al., 2011)).

In terms of spatial distribution, the Himalayan districts exhibited low concentrations of pesticides compared to the main Ganga channel, and from samples taken from artificial canals (e.g. Western Yamuna, Agra and Gurgaon canals). This is particularly the case for OCPs, such as DDT and HCH.

However, no upstream-downstream trend was detected in the Ganga and contamination levels appear to be influenced by local pollution sources and attenuate quite rapidly downstream as consequence of dilution or adsorption by river channel sediment (Narain, 2014).

High concentrations were found in the proximity of big urban agglomerates such as Delhi, Allahabad, Varanasi, and Lucknow. It has been reported that cities located along the Ganga and its tributaries have contributed to OCP pollution mainly through the release of insecticides in wastewater during vector-control campaigns (Trivedi et al., 2016).

However, the main Ganga channel is characterised by lower concentrations compared to canals, ponds, and lakes, as recorded by Singh et al. (2007) and Jit et al. (2011) in the plain between Kanpur and Lucknow. This pattern, evident in water, is less noticeable in sediment, probably due to the more limited availability of papers, which prevents any detailed spatial assessment. In addition, spatial comparisons would require accurate knowledge of the organic carbon content of the matrix, that can be highly variable in different locations. In fact, as POPs tend to be associated with organic-rich particles, sediment concentration values are influenced by organic carbon content (Binelli et al., 2007).

An overall decrease in pesticide concentration is evident in surface water, as previously reported by (Dwivedi et al., 2018). The declining trend over the 40-year time frame of the studies was evident especially for persistent pesticides, whose bans and limitations have positively affected the environmental status of the Ganga. However, no clear trend was shown for sediment. This might be due to multiple reasons, including the fewer number of papers that have studied sediment pollution and the different pollution dynamics in terms of mass load and flow rate in the two matrices.

**Figure 4:**
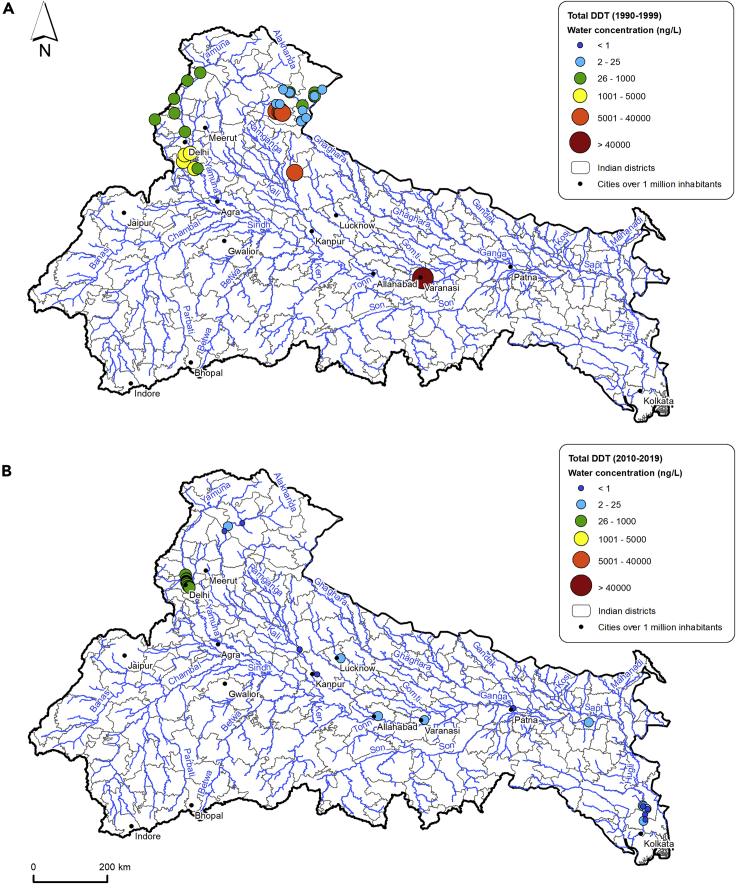
Comparison between total DDT water concentrations in the 1990s (1990–1999) and the 2010s (2010–2019) Total DDT stands for the sum of both o,o’ -and p,p’-dichlorodiphenyltrichloroethane (DDT), dichlorodiphenyldichloroethane (DDD), and dichlorodiphenyldichloroethylene (DDE).

**Figure 5:**
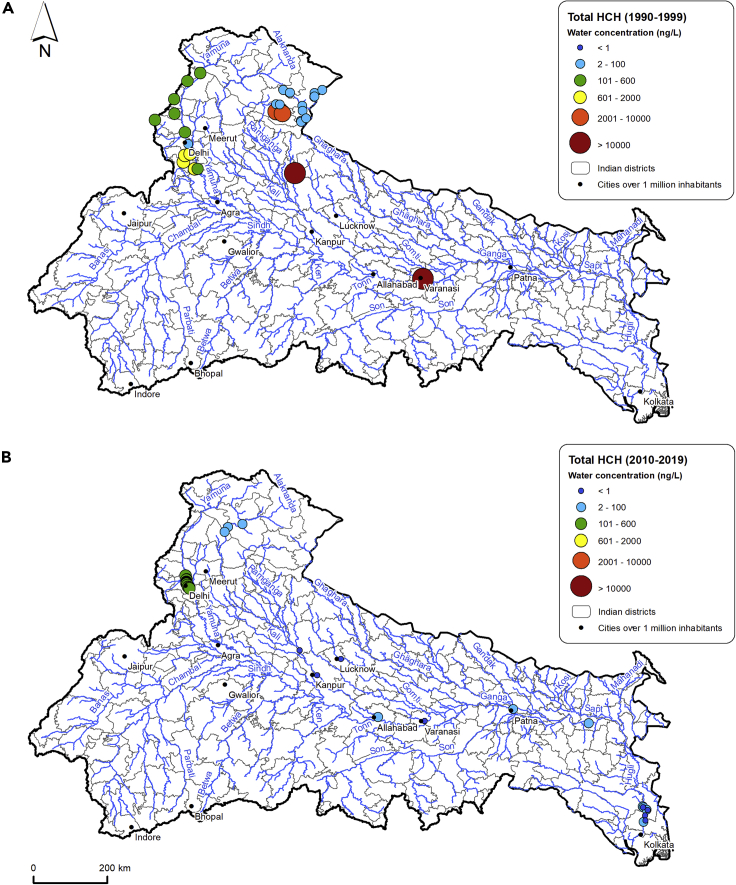
Comparison between total HCH water concentrations in the 1990s (1990–1999) and the 2010s (2010–2019) Total HCH stands for the sum of α-, β-, γ-, and δ-hexachlorocyclohexane (HCH).

Focusing on the two most studied and detected pesticides, DDT and HCH (isomers and metabolites), these showed similar spatial and temporal concentration trends. This reflects the comparable use and history of the two compounds in the region with both extensively used in agriculture since the Green Revolution but also employed for sanitation purposes after restrictions introduced in the 1990s (Yadav et al., 2015). However, there is a decrease in concentrations of both pesticides after 2010 (Figures 4 and 5).

### Industrial compounds

This category of environmental contaminants includes a variety of compounds synthesized or used in chemical plants and other manufacturing processes, or released as industrial by-products. For some of them, such as OTCs, pollution results from the disposal and breakdown of manufactured products. Unlike ECs, these contaminants have been studied in the Ganga Basin since the 1990s, or even the 1980s in the case of PAHs. These compounds are well-known for their detrimental health and environmental effects and have already been regulated by international and Indian authorities. PAHs and PCBs are now periodically monitored in India and have acceptable limits of contamination defined by the Indian drinking water quality standards (Bureau of Indian Standards (BIS, 2012)). Furthermore, in 2015 India complied with the International Convention on the Control of Harmful Anti-fouling Systems on Ships (AFS) (International Marine Organization, IMO, 2018). Most of these chemicals are included in the class of POPs listed in the Stockholm Convention, ratified by India in 2006 (UNEP, 2018). Among them are PBDEs, specifically tetra-, penta-, hexa-, hepta- and decabromodiphenyl ether, belonging to the class of brominated flame retardants (Rahman et al., 2001).

PCBs are officially recognized as POPs (UNEP, 2018). Employed in many industrial applications (e.g. transformers, capacitors, paints, and printing inks) (Erickson and Kaley, 2011), PCBs are released into the environment through leaks or fires, and spills during the transport of products containing them (S. Kumar et al., 2012).

Despite not being listed in the Stockholm Convention, many analogies can be found between PAHs and POPs, as they share lipid solubility and persistence in the environment (Abdel-Shafy and Mansour, 2016). PAHs may result from a series of combustion processes, including pyrolysis of wood to produce charcoal and carbon black, power generation from fossil fuels, incineration of waste, vehicular emissions (Malik et al., 2011). PAHs are well-known mutagens and teratogens and human carcinogens (Abdel-Shafy and Mansour, 2016).

Another common link between different classes of ICs is their ability to act as endocrine disruptors. One of the most studied examples of endocrine disruption in wildlife is imposex induced in gastropods by OTCs (Matthiessen, 2013; Sousa et al., 2014). The most notorious of these chemicals is tributyltin (TBT), a biocide used in antifouling paints, which can negatively affect non-target aquatic organisms (Bangkedphol et al., 2009; Garg et al., 2011; Ohji et al., 2007). Also PCBs have been reported as endocrine disruptors (Sharma and Kapoor, 2014).

### Location of sample points

Among the analyzed studies, 20 papers assessed ICs, with most (10 publications) reporting PAHs (Tables 5, 6, S8, and S9). As with ECs and pesticides, the geographical distribution of sample points for ICs is uneven, with most studies focused on the main channel of the Ganga, in the cities of the upper and middle reaches (e.g. Kanpur, Allahabad, Varanasi, and Patna). Besides the Ganga, studies were concentrated along the Gomti, in Delhi and the nearby districts of Haryana and Uttar Pradesh. The largest concentration of sample points is located in the Hugli reach and the deltaic region (Figure 2C), but there is no available data for south-bank rivers other than the Yamuna.

The distribution of study areas of PAHs in surface water considerably differs from sediment. The former include a greater number of papers analysing the Gomti and the Himalayan rivers, whereas in the case of sediment, more attention was given to the central area of the main channel and the deltaic region but with no data on the Himalayan reach of the Ganga. PAHs have mainly been assessed in sediment from the Hugli river and from Sundarban (Binelli et al., 2008; Guzzella et al., 2005; Zuloaga et al., 2013), in water and sediment along the Gomti, in the towns of Neemsar, Bharatpur, Lucknow, Barabanki, Sultanpur, and Jaunpur (Malik et al, 2004, 2011; Tripathi et al., 2009), and in eight cities and towns located along the Alaknanda, the Bhagirathi, and the main channel (Uttarkashi, Devprayag, Narora, Kachala, Fatehgarh, Kannauj, Kanpur, Varanasi, Patna, Farakka), as well as in the delta island of Gangasagar, and the Gangotri glacier (Ahmad et al., 1996; Sharma et al., 2018). Only one paper assessed the presence of PAHs in the Yamuna river, specifically in sediment upstream and downstream of Dehli (Agarwal et al., 2006), while one paper addressed the presence of PAHs in core samples taken in Nainital and Bhimtal Lakes, located in the Himalayan region of the basin (Choudhary and Routh, 2010).

Spatial patterns of other IC categories in surface water and sediment are similar, although many water studies were concentrated in Dehli and urban areas located along the main channel, whereas most of the papers addressing sediment were focused on the Hugli river and its estuary. PBDEs have only been investigated in sediment of the deltaic region, in Kolkata (Kwan et al., 2013) and the Sundarban wetlands (Binelli et al., 2007). PCBs have been studied in sediment of ten cities and towns located in the upper, middle, and lower reaches of the main channel (Haridwar, Kanpur, Allahabad, Buxar, Patna, Mokama, Sultanganj, Kahalgaon, Rajmahal, Farakka) (Senthilkumar et al., 1999), as well as in Delhi, Sundarban and the lower Hugli (Binelli et al., 2009; B. Kumar et al., 2012a). PCBs in river water have been investigated only along the Yamuna in NCT (B. Kumar et al., 2012b), and in irrigation canals, lakes ponds and drains in the region surrounding Delhi (S. Kumar et al., 2012). The distribution of OTCs has also been investigated in water and sediment of the Kolkata harbor, the lower Hugli and Sundarban (Antizar-Ladislao et al., 2011; Garg et al., 2011), and in water along the main channel and three minor tributaries (Loni, Pandu, and Ganda Nala), in the Kanpur-Unnao region (Ansari et al., 1998).

### Occurrence of ICs

The concentrations of industrial chemicals both in Ganga water and sediment exhibit a wide range of variability, from less than one to hundreds of ng/L and from less than one to hundreds of μg/kg, respectively. PAHs concentrations in some instances reach up to thousands of ng/L and μg/kg respectively with the highest concentrations of acenaphthylene in water (MRV: 65,850 ng/L, Lucknow (Malik et al., 2004)) and benzo[a]anthracene in sediment (MRV: 5950 μg/kg d.w., Dehli (Agarwal et al., 2006)).

The highest concentration of PBDEs in sediment was recorded for PBDE-47 (maximum value: 8.832 μg/kg d.w., Sundarban (Binelli et al., 2007)), but no measurements were made in water.

For PCBs, compounds with the highest concentration were PCB-18 in water (MRV: 314 ng/L, Delhi (S. Kumar et al., 2012)) and PCB-44 in sediment (MRV: 14.17 μg/kg d.w., Delhi (S. Kumar et al., 2012)). The highest water concentration of OTCs in water was recorded for dibutyltin (DBT, MRV: 104 ng Sn/L, Kolkata (Garg et al., 2011)), with the highest sediment concentration for TBT (MRV: 442 ng Sn/g d.w., Kolkata (Garg et al., 2011)).

With regard to the spatial distribution of ICs, no clear trend could be detected along the main Ganga channel, although Binelli et al. (2009) detected higher concentrations of PCBs in sediment within the delta region (up to 26.84 μg/kg d.w.), compared to those found ten years earlier along the main channel (ranging from 0.9 to 9.4 μg/kg d.w (Senthilkumar et al., 1999)). This was attributed to local point sources of pollution in the delta arising from urban sewage (Binelli et al., 2009)).

Sediment concentrations of OTCs detected by Garg et al. (2011) in Kolkata harbor were also one order of magnitude higher than those detected in Sundarban and the lower Hugli reach (Antizar-Ladislao et al., 2011), resulting from more limited water exchange in the harbor and direct sources of antifouling paints. In surface water, concentrations appeared to be higher in Kolkata harbor (Garg et al., 2011) than the Kanpur-Unnao region (Ansari et al., 1998) and shows that despite the gradual decrease in organotin-based paints, contamination levels were high until recently. Moreover, Kolkata is a contamination hotspot of PBDEs (Kwan et al., 2013).

For PAHs in the Hugli reach, concentrations reported by Zuloaga et al. (2013) were systematically higher than those found by Guzzella et al. (2005) that were below detection limits at most sites. This increasing trend is attributed to the presence of local point inputs from industrial sources and other combustion processes (Zuloaga et al., 2013). All the studies on the Gomti, however (Malik et al, 2004, 2011; Tripathi et al., 2009), reported comparable although highly variable concentrations, with sediment-associated total PAHs ranging from 50 μg/kg d.w. to more than 3000 μg/kg d.w. Surprisingly high concentrations of total PAHs were detected by in the Himalayan region of Nainital lakes, attributed to frequent forest fires and the use of coal and wood for heating and cooking purposes (Choudhary and Routh, 2010). This would appear to be a recent phenomenon as publications in the mid-1990s recorded very low PAH concentrations (Ahmad et al., 1996).

## Health and environmental risks of organic contaminants in the Ganga compared with other river systems in India and worldwide

### Emerging contaminants

Sharma et al. (2019) reported maximum concentrations of many PPCPs (e.g. the NSAIDs diclofenac, ibuprofen, naproxen) lower than those found in Kaveri, Vellar and Tamiraparani rivers in peninsular India (Shanmugam et al., 2014), which are similar or even lower than those detected by Mutiyar et al. (2018) in NCT and by Williams et al. (2019) in Udaipur (Ahar river). Concentrations of ciprofloxacin in the Ganga were up to six orders of magnitude lower than those found in the Isakavagu-Nakkavagu rivers in Hyderabad (Fick et al., 2009). Similarly, values of triclosan reported from southern India, in the Tamiraparani, Kaveri and Vellar rivers (Ramaswamy et al., 2011), were higher than those found in the Ganga but comparable to those found by Nag et al. (2018) in the Gomti. Reference values are reported in Table S1.

At present it is not possible to determine the impact of these concentration levels on ecosystem health because of the lack of official regulation or guidelines for ECs. Nevertheless, ecological risk assessments performed by Sharma et al. (2019) and Mutiyar et al. (2018) demonstrated that the detected values of PPCPs along the main Ganga channel and in Delhi posed no significant human health concern, although there was a moderate risk for aquatic organisms (algae and *Daphnia magna*) associated with some of the PPCPs. Neither the most detected PPCPs (i.e. acetaminophen, carbamazepine, ibuprofen, ketoprofen, sulfamethoxazole and DEET) nor caffeine, whose concentrations were the highest recorded among ECs, exceeded the predicted no-effect concentrations for invertebrates and fish summarized by Sharma et al. (2019) and Mutiyar et al. (2018). However, caffeine, sulfamethoxazole and triclosan exceeded the PNECs calculated for algae (15, 27 and 1.4 ng/L, respectively).

According to Mutiyar et al. (2018), antibiotic residues detected in Delhi were concentrations shown to cause acute toxicity; in particular, maximum concentrations of ciprofloxacin (1190 ng/L) approached those causing growth inhibition to algae. It remains to be seen whether these compounds exhibit synergic effects in case of exposure to multiple active substances. Besides acute toxicity, antibiotics pose the risk of antimicrobial resistance, which has been extensively recorded in bacterial isolates recovered from Indian surface waters, including the Gomti, the Yamuna and the Kshipra rivers (Diwan et al., 2018; Philip et al., 2018). In the case of triclosan, all the concentrations reported by Nag et al. (2018), despite posing no risk for human health, exceeded the Canadian Federal Environmental Quality Guideline (FEQG, 0.47 μg/L, Table S3), a reference value expressing the likelihood of direct adverse effects on aquatic life. For PFAS, according to Yeung et al. (2009) river water concentrations from India appeared to be lower than those reported for other Asian countries. In India, higher concentrations of PFOS and PFOA were found in the Cooum River in Chennai and in Sri Lanka (Table S1). Sediment concentrations of PFOS recorded in the Hugli estuary were low, being below the detection limit in all the sampling stations. PFOA concentrations are however comparable to those recorded in river estuaries of the Bohai Bay, China, and one-two orders of magnitude higher than those recorded in Vietnam (Lam et al., 2017). PFOA and PFOS were below the WHO drinking water guidelines (4 and 0.4 μg/L respectively, Table S3), and PFOS was below the Canadian FEQG (6.8 μg/L, Table S3). With reference to other classes of ECs, the sediment concentration of phthalates analyzed in the Gomti were lower in comparison to the values recorded in China and Taiwan (especially di-(2-ethylhexyl) phthalate (Srivastava et al., 2010)), while water concentrations of benzotriazole and bisphenol A were comparable to those found in other Asian countries (hundreds of ng/L (Williams et al., 2019)). Bisphenol A was below the Canadian FEQG (3.5 μg/L, Table S3).

### Pesticides

Despite multiple restrictions of pesticide use (Agarwal et al., 2015) very high levels of pesticides are still detected in the Ganga basin. Mondal et al. (2018) reported residues of α-, β-, and δ-HCH and p,p’-DDT exceeding the EC limit in drinking water (0.10 μg/L for single pesticide, Table S3) in 56.2 and 100 per cent of samples collected in river water samples of the delta region, while only 6.2 and 12.5 per cent of samples were above the EC limit for the 16 detected pesticides (DDT and metabolites, HCH isomers, endosulfan isomers, methylparathion, monocrotophos, phorate, buthaclor). Concentrations exceeding EC drinking water limits have also been reported along the Ganga, in Allahabad (Raghuvanshi et al., 2014) and the Hugli (Mondal et al., 2018). In the case of pond water collected in the delta region, the EC limit was exceeded in 25 per cent of methyl parathion, 31.2 per cent of chlorpyrifos, and 6.2 per cent of phorate and atrazine samples (Mondal et al., 2018). Even higher water levels of α-HCH, α-endosulfan, dicofol, heptachlor, p,p’-DDE (dichlorodiphenyldichloroethylene), alachlor and butachlor were found by (Trivedi et al., 2016) in the Gomti river. Concentrations of α-HCH, aldrin and endosulfan found by (Mutiyar and Mittal, 2013) in Uttar Pradesh and Bihar exceeded the Indian drinking water quality standards (Table S3). Based on the assessment conducted by Mondal et al. (2018), persistent OC pesticides such as HCH isomers, DDT isomers and metabolites and endosulfan, still pose a potential risk to freshwater aquatic animals and invertebrates.

### Industrial compounds

Malik et al. (2011) found that total PAH concentrations in water and sediment of the Gomti are higher than in other Asian rivers such as the Gao-ping in Taiwan, the Yellow River, and the Qiantang in China. However, the levels of PAHs in the Gomti appeared to be considerably lower than that reported in the Jinsha river of China. Choudhary and Routh (2010), who assessed the impact of PAH pollution in sediment, found that fluorene, acenaphthylene, and total PAH concentrations exceeded the toxicological endpoints for aquatic fauna even in the Himalayan districts of Nainital and Bhimtal. Similarly, Zuloaga et al. (2013), who analyzed the distribution of PAHs in the sediment of Sundarban wetlands, reported possible biological impact associated with the recorded levels of pollutants. PBDEs sediment concentrations, especially those recorded in Kolkata, are comparable to the PBDE levels found in other Asian areas (Binelli et al., 2007).

Maximum sediment concentrations of PCBs recorded in Sundarban and Delhi were higher than those detected in the west coast of Sri Lanka (Rajendran et al., 2005) and in the southern part of the Bay of Bengal (Guruge and Tanabe, 2001), whereas water concentrations were comparable to those found in coastal waters of Daya Bay, China (Zhou et al., 2001). OTC concentrations reported in the Ganga basin were generally lower than those of the coastal areas of Thailand and India (in particular TBT) and in the sediment of the Zuari estuary, located on the west coast of India (Garg and Bhosle, 2005; Harino et al., 2006). Garg et al. (2011), however, observed that concentrations of TBT compounds in water at all the sampling sites in Kolkata were higher than those known to induce imposex in gastropods (<10 ng/L). Reference values are reported in Table S2.

Total PCB water values recorded in the Ganga basin were above both the Indian Drinking Water Guidelines and the US EPA Water Quality Criteria (WQC, 0.5 and 0.14 μg/L for acute and chronic toxic effects on biota) (Table S3). PCB levels in sediment were all below the Canadian Sediment Quality Guideline (CSQG) for the protection of aquatic life (34.1 μg/kg for total PCBs, Table S3).

Concentrations of OTCs and TBT in water were all below the US WQC for both acute and chronic toxic effects on biota (0.46 and 0.072 μg/L, respectively, Table S3). But at many sites TBT exceeded the European Environmental Quality Standards, both the Annual Average (AA, 0.0002 μg/L) and the Maximum Allowable Concentration (MAC, 0.0015 μg/L), representing concentrations considered to protect the living environment against chronic and acute toxicity, respectively (Table S3). TBT concentration in sediment was higher than the upper guideline of the Australian Sediment Quality Guideline Values for TBT (70 ng Sn/g, corresponding to around 29 μg/kg).

## Research gaps and basin-scale implications

The review of 61 papers addressing surface water and sediment pollution revealed the presence of numerous knowledge gaps, the identification of which is essential for guiding future research campaigns and risk assessments.

One of the most critical research gaps is the lack of basin-scale assessments. No studies of this kind have been published either for ECs or ICs, whereas the first catchment review of pesticides was undertaken in 2013 (Mutiyar and Mittal, 2013). All earlier publications monitored either specific tributaries or the main channel, occasionally including canals or minor rivers. Given the size of the catchment and significant hydrologic, demographic and environmental variability in the region, only basin-scale studies will allow an understanding of the impact of tributaries on pollution patterns.

Further research is required also to understand the variability of concentrations according to the season (dry, winter and monsoon season) and the flooding regime. While only 13 papers (Tables S4–S9) provided an analysis of the seasonal variability of concentrations according to the river flow, studies of this kind would help the risk assessment by identifying the time frames in which higher concentrations are detected.

In the case of ECs, the gaps are exacerbated by the limited number of available studies, with only three publications focused on the cities along the Ganga main channel (Sharma et al, 2016, 2019; Yeung et al., 2009) and three on the NCT in the Yamuna sub-catchment (Mutiyar et al., 2018; Mutiyar and Mittal, 2012, 2014a), where the high population density is likely to constitute a direct source of PPCP contamination from domestic effluents. Although PPCPs and PFAS concentrations in the main channel were generally below 10 ng/L, and often close to detection limits, the widespread use of these compounds in densely populated areas, and their detection at higher concentrations in other regions of India (Philip et al., 2018), justify the necessity of further studies addressing the topic. Considering a resource-limited scenario, the most frequently detected compounds, such as acetaminophen, carbamazepine, ibuprofen, ketoprofen, sulfamethoxazole, DEET and caffeine, should be prioritized. Also antibiotics and antibiotic resistance, representing a major challenge for human health, should be further investigated.

Another knowledge gap is the lack of studies on pesticides other than OCPs and OPhs. While OCPs, for the most part, can be considered legacy compounds, no publications are available on carbamates and only one on pyrethroids, that include many active substances currently used in India (Center for Science and Environment, 2013). Besides insecticides, also herbicides and fungicides have been poorly investigated.

## Conclusions

This review demonstrates that data on organic contaminants in the Ganga basin is still fragmentary and mainly focused on the main channel, the Yamuna, the Gomti and the delta region.

The most studied organic contaminants were OCPs, followed by OPhs and PCBs. With reference to ECs, the investigation of PPCPs has been particularly neglected in sediment, but widely investigated in the case of ICs and, to a lesser extent, pesticides. Although pesticide concentrations decreased between 1980 and 2019 as a result of restriction in their use, higher concentrations were reported for PCBs and OTCs in the last decade. Recently hotspots of contamination have emerged within and downstream of many of the large urban areas such as Delhi, Kanpur, Allahabad, Varanasi, Patna, Kolkata, along the Gomti and in the Sundarban Wetlands. In these locations high levels of all categories of pollutants have been reported with domestic and industrial effluents as major contributors to pollution. Even pesticides, whose main source is agriculture, were often reported in association with urban wastewater, since the two most studied insecticides, DDT and HCH, have long been utilised for sanitation purposes in the region.

We recommend that future assessments should prioritize investigating ECs, especially PPCPs. For pesticides, more focus is required for herbicides and carbamate insecticides that hitherto have not been fully investigated. The seasonal variability of organic contaminants especially in relation to flooding regime needs also to be studied.

The primary knowledge gap is a catchment-scale understanding of organic contaminant dispersal and storage, including tributary contributions and downstream attenuation patterns in the main Ganga channel. This is urgently needed for effective pollution control, watershed management and the protection of human and ecosystem health.

## Methods

All methods can be found in the accompanying Transparent methods supplemental file.
