# Supplementary material for: Organic contaminants in Ganga basin: from the Green Revolution to the emerging concerns of modern India
Source: iScience. 2021 Feb 3;24(3):102122. doi: 10.1016/j.isci.2021.102122 (PMC7900350; doi:10.1016/j.isci.2021.102122)
Supplement: Document S1. Figure S1 and Tables S1–S3 and S10 [file mmc1.pdf]

iScience, Volume *24*

Supplemental Information

Organic contaminants in Ganga basin: from the Green Revolution to the emerging concerns of modern India

Aurora Ghirardelli, Paolo Tarolli, Mangalaa Kameswari Rajasekaran, Amogh Mudbhatkal, Mark G. Macklin, and Roberta Masin

Supplemental Information

Supplemental data items

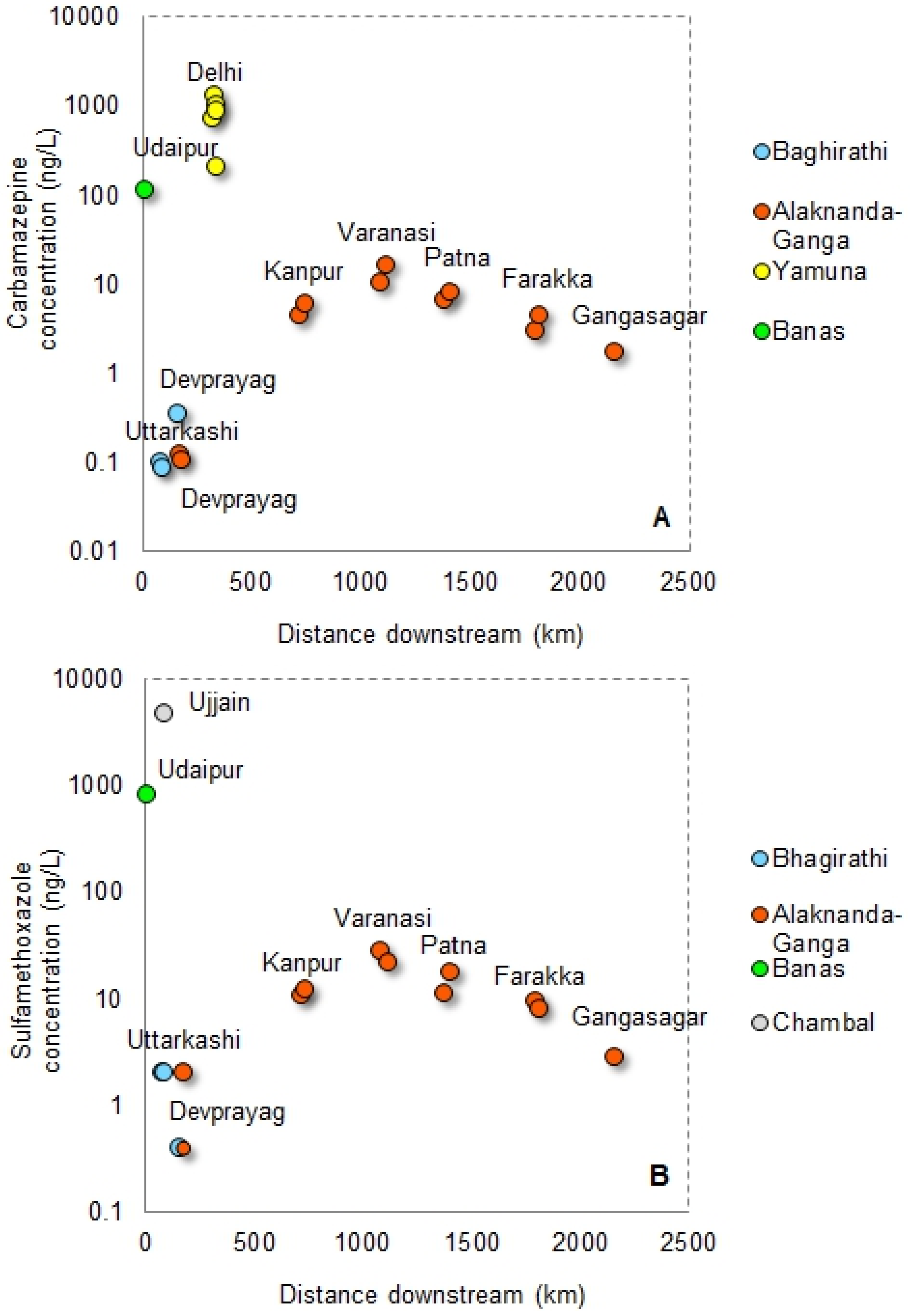

Figure S1. Maximum water concentrations of two selected ECs along the main channel (Alaknanda-Ganga) and the main tributaries. Related to paragraph 4.2.

(A) Carbamazepine; (B) Sulfamethoxazole. The distance downstream of the source was calculated along the Ganga, the Yamuna, the Chambal and the Banas, even though Ujjain and Udaipur are located on the banks of minor sub-tributaries of Chambal and Banas respectively. For the main channel and the Himalayan rivers, a distinction was made between the longest path, which included the Alaknanda and the Ganga itself, and the Bhagirathi, which joins the Alaknanda in Devprayag.

Table S1. Concentrations of emerging contaminants from other regions of India and other Asian countries. **Related to paragraph 7.1.**
Comparison with contamination levels detected in surface water and sediment of the Ganga basin. Abbreviations are listed in **table S10**.

**Compound**	**Concentration range Ganga basin (ng/L)**		**Water concentrations (ng/L)**		**Location**	**Reference**
	**Min**	**Max**	**Min**	**Max**		
Ciprofloxacin	BDL	1440	10,000	2,500,000	Isakavagu and Nakkavagu Rivers, Telangana (India)	(Fick et al., 2009)
Diclofenac	BDL	412	BDL	106	Kaveri, Vellar, and Tamiraparani rivers, Tamil Nadu (India)	(Shanmugam et al., 2014)
Ibuprofen	BDL	2302	BDL	200	Kaveri, Vellar, and Tamiraparani rivers, Tamil Nadu (India)	(Shanmugam et al., 2014)
Ketoprofen	BDL	107	BDL	100	Kaveri, Vellar, and Tamiraparani rivers, Tamil Nadu (India)	(Shanmugam et al., 2014)
Caffeine	BDL	37,476	36,670	46,970	Pili and Nag rivers, Maharastra (India)	(Shanmugam et al., 2014)
Triclosan	BDL	9650		944	Tamiraparani river, Tamil Nadu (India)	(Ramaswamy et al., 2011)
Triclosan			3800	5160	Kaveri and Vellar rivers, Tamil Nadu (India)	(Ramaswamy et al., 2011)
PFOS	BDL	0.85	-	3.91	Cooum River, Tamil Nadu (India)	(Yeung et al., 2009)
PFOS			-	2.7	Kelani River, Sri Lanka	(Yeung et al., 2009)
PFOA	BDL	1.18	-	23.1	Cooum River, Tamil Nadu (India)	(Yeung et al., 2009)
PFOA			-	1.7	Kelani River, Sri Lanka	(Yeung et al., 2009)
Benzotriazole	BDL	526	15	5850	Asian Countries	(Williams et al., 2019)
Bisphenol A	BDL	299	35	167	Asian Countries	(Williams et al., 2019)
**Compound**	**Concentration range Ganga basin (µg/kg d.w.)**		**Sediment concentrations (µg/kg d.w.)**		**Location**	**Referenceµg/kg**
	**Min**	**Max**	**Min**	**Max**		
PFOA	BDL	14.09	<0.02	29.02	Bohai Bay (China)	(Lam et al., 2017)
PFOA			< 0.20	-	Vietnam	(Lam et al., 2017)
DEHP	BDL	324	500	23,900	Klang River, Malaysia	(Srivastava et al., 2010)
DEHP			-	1630	Taiwan	(Srivastava et al., 2010)

Table S2. Concentrations of industrial compounds from other regions of India and other Asian countries. Related to paragraph 7.2.
Comparison with contamination levels detected in surface water and sediment of the Ganga basin. Abbreviations are listed in **table S10**.

**Compound**	**Concentration range Ganga basin (ng/L)**		**Water concentrations (ng/L)**		**Location**	**Reference**
	**Min**	**Max**	**Min**	**Max**		
Total PCBs	0.18	1768	91.1	231.8	Daya Bay (China)	(Zhou et al., 2001)
TBT	BDL	9.41	-	1246	Coastal areas of Thailand	(Garg and Bhosle, 2005)
TBT			-	2800	Coastal areas of India	(Harino et al., 2006)
Total PAHs	0.05	81,400	70.3	1844.4	Qiantang River, China	(Chen et al., 2007)
Total PAHs			10	9400	Gao-Ping, Taiwan	(Doong and Lin, 2004)
Total PAHs			21.8	497	Yellow River, China	(Wang et al., 2008)
**Compound**	**Concentration range Ganga basin (µg/kg d.w.)**		**Sediment concentrations (µg/kg d.w.)**		**Location**	**Reference**
	**Min**	**Max**	**Min**	**Max**		
Total PBDEs	0.08	57.1	0.01	59	Asian Countries	(Binelli et al., 2007)
Total PCBs	0.18	26.84	0.45	4.4	West Coast of Sri Lanka	(Rajendran et al., 2005)
Total PCBs			0.023	6.57	Bay of Bengal	(Guruge and Tanabe, 2001)
TBT	BDL	1080	-	670	Zuari estuary, Goa (India)	(Meena et al., 2009)
Total PAHs	9.18	217,000	91.3	1835.2	Qiantang River, China	(Chen et al., 2007)
Total PAHs			1.43	356.0	Gao-Ping, Taiwan	(Doong and Lin, 2004)

Table S3. Guidelines and advisory levels in water and sediment for selected emerging contaminants, pesticides and industrial compounds. Related to chapter 7 and tables 1-6.
Abbreviations are listed in **table S10**.

**Compounds**	**Guideline references**	**Water concentrations (ng/L)**	
Triclosan	Canadian Federal Environmental Quality Guideline (Government of Canada, 2019)	470	
PFOA	WHO Drinking Water Guidelines (WHO, 2017)	4000	
PFOS	WHO Drinking Water Guidelines (WHO, 2017)	400	
PFOS	Canadian Federal Environmental Quality Guideline (Government of Canada, 2019)	6800	
Bisphenol A	Canadian Federal Environmental Quality Guideline (Government of Canada, 2019)	3500	
Active substances in pesticides	European Union Groundwater Quality Standard (EC, 2006)	100	
α-HCH	Indian drinking water quality standards (BIS, 2012)	10	
Aldrin	Indian drinking water quality standards (BIS, 2012)	30	
endosulfan	Indian drinking water quality standards (BIS, 2012)	400	
Total PCBs	Indian drinking water quality standards (BIS, 2012)	5000	
Total PCBs	US EPA Water Quality Criteria (USEPA, 2018)	14 [a]	500 [b]
TBT	US EPA Water Quality Criteria (USEPA, 2018)	72 [a]	470 [b]
TBT	European Environmental Quality Standards (European Commission, 2013)	0.2 [c]	1.5 [d]
**Compounds**	**Guideline references**	**Sediment concentrations µg/kg d.w.**	
Total PCBs	Canadian Sediment Quality Guideline for the protection of aquatic life (Canadian Council of Ministers and of the Environment, 2018)	34.1	
TBT	Australian Sediment Quality Guideline Values (Simpson et al., 2013)	70 ng Sn/g	

[a] AA = Annual Average (chronic toxicity); [b] CCC = Criterion Continuous Concentration (chronic toxicity); [c] CMC = Criterion Maximum Concentration (acute toxicity); [d] MAC = Maximum Allowable Concentration (acute toxicity).

Table S10. List of acronyms and abbreviations reported in the tables. Related to tables 1-6 and tables S1-9.

Acronym	Meaning
2,4 D	2,4-dichlorophenoxyacetic acid
BDL	Below detection limit
d.w.	Dry Weight
DBT	Dibutyltin
DDE	Dichlorodiphenildichloroethilene
DDT	Dichlorodiphenyltrichloroethane
DEET	Dietiltoluamide
DEHA	Bis (2-ethylhexyl) adipate
DEHP	Di-(2-ethylhexyl) phthalate
HCB	Hexachlorobenzene
HCH	Hexachloro cyclohexane
MBT	Monobutyltin
NSAIDs	Nonsteroidal anti-inflammatory drugs
OCPs	Organochlorine pesticides
OPhs	Organophosphates
OTCs	Organotin compounds
PAHs	Polycicyclic aromatic hydrocarbons
PBDE(s)	Polybrominated diphenyl ether(s)
PCB(s)	Polychlorinated biphenyl(s)
PFAS	Per- and Polyfluoroalkyl substances
PFBS	Perfluorobutane sulfonate
PFHXA	Perfluorohexanoic acid
PFOA	Perfluorooctanoic acid
PFOS	Perfluorooctane sulfonate
TBT	Tributyltin
US EPA	United States Environmental Protection Agency
WHO	World Health Organisation

Supplemental References

Canadian Council of Ministers, of the Environment, 2018. Canadian Environmental Quality Guidelines for the Protection of Aquatic Life [WWW Document]. URL http://st-ts.ccme.ca/en/index.html (accessed 1.17.19).

Chen, Y., Zhu, L., Zhou, R., 2007. Characterization and distribution of polycyclic aromatic hydrocarbon in surface water and sediment from Qiantang River, China. J. Hazard. Mater. 141, 148–155. https://doi.org/10.1016/j.jhazmat.2006.06.106

Doong, R., Lin, Y., 2004. Characterization and distribution of polycyclic aromatic hydrocarbon contaminations in surface sediment and water from Gao-ping River, Taiwan. Water Res. 38, 1733–1744. https://doi.org/10.1016/j.watres.2003.12.042

EC, 2006. Directive 2006/118/EC of the european parliament and of the council of 12 december 2006 on the protection of groundwater against pollution and deterioration. [WWW Document]. Off. J. Eur. Union. URL https://eur-lex.europa.eu/LexUriServ/LexUriServ.do?uri=OJ:L:2006:372:0019:0031:EN:PDF

European Commission, 2013. Directive 2013/39/EU of the European Parliament and of the Council of 12 August 2013 amending Directives 2000/60/EC and 2008/105/EC as regards priority substances in the field of water policy, Official Journal of the European Union.

Government of Canada, 2019. Federal Environmental Quality Guidelines (FEQGs) [WWW Document]. URL https://www.canada.ca/en/health-canada/services/chemical-substances/fact-sheets/federal-environmental-quality-guidelines.html (accessed 1.16.19).

Guruge, K.S., Tanabe, S., 2001. Contamination by Persistent Organochlorines and Butyltin Compounds in the West Coast of Sri Lanka. Mar. Pollut. Bull. 42, 179–186. https://doi.org/10.1016/S0025-326X(00)00140-5

Harino, H., Ohji, M., Wattayakorn, G., Arai, T., Rungsupa, S., Miyazaki, N., 2006. Occurrence of Antifouling Biocides in Sediment and Green Mussels from Thailand. Arch. Environ. Contam. Toxicol. 51, 400–407. https://doi.org/10.1007/s00244-005-0246-x

Lam, N.H., Cho, C.-R., Kannan, K., Cho, H.-S., 2017. A nationwide survey of perfluorinated alkyl substances in waters, sediment and biota collected from aquatic environment in Vietnam: Distributions and bioconcentration profiles. J. Hazard. Mater. 323, 116–127. https://doi.org/10.1016/J.JHAZMAT.2016.04.010

Meena, R.M., Garg, A., Jadhav, S., 2009. Seasonal Variation in Organotins in the Waters of the Dona Paula Bay, West Coast of India. Bull. Environ. Contam. Toxicol. 82, 586–589. https://doi.org/10.1007/s00128-009-9634-7

Rajendran, R.B., Imagawa, T., Tao, H., Ramesh, R., 2005. Distribution of PCBs, HCHs and DDTs, and their ecotoxicological implications in Bay of Bengal, India. Environ. Int. 31, 503–512. https://doi.org/10.1016/J.ENVINT.2004.10.009

Simpson, S.L., Batley, G.E., Chariton, A.A., 2013. Revision of the ANZECC/ARMCANZ sediment quality guidelines. CSIRO Land and Water Science Report 08/07.

USEPA, 2018. Current Water Quality Criteria Tables [WWW Document]. Water Qual. Criteria . URL https://www.epa.gov/wqc (accessed 11.30.18).

Wang, J.-Z., Nie, Y.-F., Luo, X.-L., Zeng, E.Y., 2008. Occurrence and phase distribution of polycyclic aromatic hydrocarbons in riverine runoff of the Pearl River Delta, China. Mar. Pollut. Bull. 57, 767–774. https://doi.org/10.1016/j.marpolbul.2008.01.007

WHO, 2017. Guidelines for drinking-water quality, 4th edition, incorporating the 1st addendum.

Zhou, J.L., Maskaoui, K., Qiu, Y.W., Hong, H.S., Wang, Z.D., 2001. Polychlorinated biphenyl congeners and organochlorine insecticides in the water column and sediments of Daya Bay, China. Environ. Pollut. 113, 373–384. https://doi.org/10.1016/S0269-7491(00)00180-9
